# Acute myocarditis complicated with permanent complete atrioventricular block caused by *Escherichia coli* bacteremia: A rare case report

**DOI:** 10.1097/MD.0000000000017833

**Published:** 2019-11-01

**Authors:** Ching-Tsai Hsu, Po-Jen Hsiao, Ching-Han Liu, Yen-Lien Chou, Bo-Hau Chen, Jun-Ting Liou

**Affiliations:** a Division of Cardiology, Department of Internal Medicine, Tri-Service General Hospital, National Defense Medical Center, Taipei; b Division of Cardiology, Department of Internal Medicine, Taoyuan Armed Forces General Hospital, Taoyuan; c Division of Nephrology, Department of Internal Medicine, Taoyuan Armed Forces General Hospital, Taoyuan; d Division of Nephrology, Department of Internal Medicine, Tri-Service General Hospital, National Defense Medical Center, Taipei; e Department of Life Sciences, National Central University, Taoyuan City; f Division of Nephrology, Department of Medicine, Fu Jen Catholic University Hospital, School of Medicine, Fu Jen Catholic University, New Taipei City; g Division of Cardiology, Department of Internal Medicine, Kaohsiung Armed Forces General Hospital, Kaohsiung; h Department of Pediatrics, Taoyuan Armed Forces General Hospital, Taoyuan, Taiwan.

**Keywords:** acute myocardial infarction, acute myocarditis, complete atrioventricular block, *E coli* septic shock

## Abstract

**Rationale:**

Acute myocarditis complicated with complete atrioventricular block (CAVB) is rare in clinical scenario. We report an uncommon case of myocarditis complicated with permanent CAVB caused by *Escherichia coli* (*E coli*) bacteremia.

**Patient concerns:**

A 77-year-old woman presented at the emergency department with chest pain, dizziness, nausea, and cold sweats of 1-day duration. She had histories of type 2 diabetes mellitus, hyperlipidemia, and chronic kidney disease with regular medical therapy.

**Diagnosis:**

Both blood and urine cultures were positive for *E coli*. Regional inferior wall motion abnormalities on echocardiography, unexplained life-threatening arrhythmias, newly abnormal electrocardiogram, elevated cardiac troponins, and healthy coronary arteries on angiography were consistent with *E coli*-induced myocarditis.

**Interventions:**

The patient received implantation of a dual-chamber pacemaker because of irreversible CAVB.

**Outcomes:**

The patient was discharged on day 8 and remained asymptomatic at 15 months of follow-up, with ST-segment normalization and normal left ventricular function.

**Lessons:**

This extremely rare case of E coli-induced myocarditis masquerading as acute STEMI and with permanent CAVB sequelae, highlights the importance of sensitivity to non-ischemia etiologies of ST-segment elevation and the potential impact of E coli sepsis on the cardiac conduction system.

## 1 Introduction

Most cases of acute myocarditis are caused by viruses or bacteria, but *Escherichia coli (E coli*) has very rarely been identified as a cause.^[1,2,3]^ Myocarditis is characterized by abnormal cardiac biomarkers, systolic dysfunction, and conduction abnormalities. The symptoms are similar to those of acute coronary syndrome, and the electrocardiogram may mimic that observed in ST-elevation myocardial infarction (STEMI).^[1,4,5]^ The initial symptoms of *E coli* myocardial infection may be subtle, and if neglected can lead to catastrophe.

## 2 Case report

**Figure 1:**
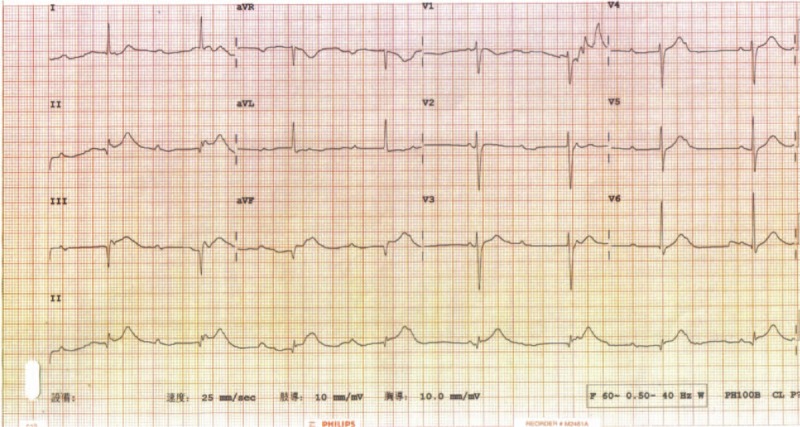
Twelve-lead electrocardiography shows ST-segment elevation in leads II, III, and aVF with complete atrioventricular block.

**Figure 2:**
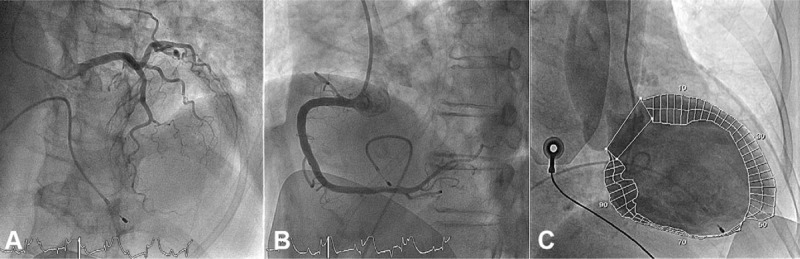
Coronary angiography and a left ventricular angiogram show normal epicardial coronary arteries (A and B) and akinesia at the mid-to-apical inferior wall of the left ventricle (C).

A 77-year-old woman presented at our emergency department room with chest pain, dizziness, nausea, and cold sweats of 1-day duration. She reported histories of type 2 diabetes mellitus, hyperlipidemia, and chronic kidney disease with regular treatment at a local medical clinic. She presented with a fever of 39.1°C, bradycardia with a heart rate of 49 bpm, and a blood pressure of 77/50 mmHg. Laboratory values included a white blood cell count, 14,810 cells/mm^[3]^; band neutrophils, 3.8%; Creatinine, 2.1 mg/dL; creatine phosphokinase, 137 U/L; troponin I, 6.07 ng/mL and C-reactive protein, 32.27 mg/dL. Serum electrolyte levels and liver function were within normal limits. Urinalysis results were significant for microhematuria with 2+ blood and 75 to 100 WBCs per high-power field. The nitrite test was positive. The electrocardiogram (ECG) revealed ST-segment elevation in leads II, III, and aVF and a complete atrioventricular block (CAVB, Fig. 1). Plain chest radiography revealed a normal heart size. Beside transthoracic echocardiography demonstrated a normal heart structure, preserved left ventricular systolic function, and an estimated ejection fraction of 50%. The initial diagnosis was urinary tract infection with septic shock. The patient was treated by aggressive fluid resuscitation, high dose inotropic agents, and antibiotics (ceftriaxone). However, consideration of the laboratory values, patient symptoms, and ECG finds led to suspicion of acute inferior wall STEMI complicated by CAVB. Temporary transvenous pacing was initiated. Emergent coronary angiography (CAG) revealed completely normal coronary arteries (Fig. 2A, B) and a left ventricular angiogram disclosed akinesia at the mid-to-apical inferior wall of the left ventricle and a left ventricular ejection fraction of 50% (Fig. 2C). The patient's pallor, dizziness, diaphoresis, and vague chest tightness were relieved by transvenous cardiac pacing at a rate of >70 bpm. The symptoms were probably the result of CAVB with bradycardia and low cardiac output. The patient was transferred to the cardiac care unit because of unstable hemodynamics.

Blood and urine cultures were positive for *E coli*. The patient's blood pressure stabilized, leukocytosis resolved, and the cardiac enzymes decreased with antibiotic treatment. Despite significant clinical improvement on day 4 after admission, intermittent CAVB and dizziness persisted. A dual-chamber pacemaker was implanted because of the possibility of irreversible CAVB. The patient was discharged 8 days after admission after an uneventful postoperative course. Evaluation of her pacemaker 15 months after implantation showed atrial pacing of 27% and ventricular pacing of 70%, with evidence of a sequela of irreversible conduction abnormalities. She remained asymptomatic with normalization of ST segments and left ventricular function.

## 3 Discussion

ST-segment elevation frequently occurs in acute myocardial infarction. In patients presenting with symptoms suggestive of myocardial ischemia, ST-segment elevation is considered to reflect acute transmural myocardial ischemia and/or myocardial damage caused by thrombotic occlusion of an epicardial coronary artery. Elevated cardiac biomarkers, ECG abnormalities, and chest pain help to diagnose acute coronary syndrome, but may also occur in patients with normal coronary arteries. Sepsis-induced myocardial ischemia and ST-segment elevation are rare, but may cause myocyte damage resulting in abnormal electrical activity including ST and T wave changes, ST elevation, atrial and ventricular arrhythmias, atrial-ventricular and intraventricular conduction defects, and variant early repolarization.^[6]^ The European Society of Cardiology has described the differential diagnosis of myocardial infarction symptoms in the absence of coronary artery obstruction.^[5]^ The noncoronary causes of elevated troponin including myocarditis, Takotsubo cardiomyopathy, pulmonary embolism, and sepsis should be considered.^[5]^ The clinical presentation and diagnostic characteristics of this patient, which included regional inferior wall motion abnormalities on echocardiography, unexplained life-threatening arrhythmias, new abnormal ECG changes, and a >50% elevation of cardiac troponins in the absence of coronary stenosis, were consistent with myocarditis.

Myocarditis can be caused *Staphylococcus aureus*, *Streptococcus*, *Pneumococcus*, *Neisseria meningitides*, and other bacteria.^[2,3]^*E coli* has not rarely been reported as a cause of myocarditis. The eight previous reports of *E coli*-induced myocarditis published between 1980 and 2018 and retrieved from PubMed are summarized in Table 1.^[1–4,7–9]^ Histopathologically confirmed *E coli*-induced myocarditis is rare. Two previously reported cases included descriptions of necropsy tissue. Both found extensive neutrophil infiltration of the myocardium and abscesses adjacent to the atrioventricular node. Both findings supported a diagnosis of bacterial myocarditis.^[3,7]^ Most of the previously reported cases had ECG changes suggestive of myocardial ischemia with a normal CAG and were accompanied by urinary tract infection by *E coli*. In this patient, blood and urine cultures were positive for *E coli*, suggesting that a urinary tract infection might have been the cause of myocarditis. *E coli*-induced myocarditis masquerading as acute STEMI with subsequent development of permanent CAVB is extremely rare.

**Table 1:**
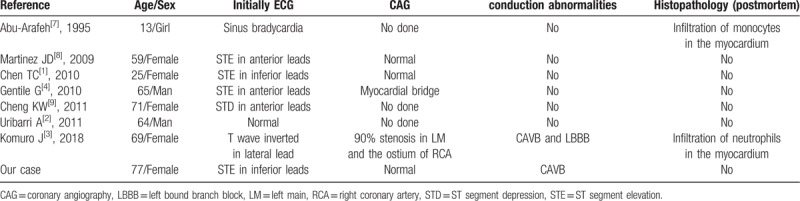
Cases of *E coli*-induced bacterial myocarditis.

It is not clear how bacterial sepsis can cause myocarditis. *E coli* is a cause of many Gram-negative extraintestinal infections and is a common cause of bacteremia.^[2]^ Bacterial peptidoglycan-associated lipoprotein (PAL) is an outer-membrane protein present only in GNB,^[10]^ and was released into the bloodstream in an mouse model of *E coli* sepsis.^[11]^ PAL binding to toll-like receptor 2 induces cardiomyocyte dysfunction and inflammatory responses mediated by tumor necrosis factor (TNF)-α.^[10]^ Another possible mechanism involves lipopolysaccharide-induced production of TNF-α, interleukin-6 and nitric oxide.^[2,4,11,12]^ The resulting inflammation and signaling following binding to toll-like receptor 4 could result in cardiomyocyte damage sufficient to cause myocarditis.^[2,12,13]^

In conclusion, the management of this patient with *E coli* infection illustrates the importance of assessing cardiac status in patients with sepsis given the potential for cardiac complications such as heart failure and severe conduction abnormalities. It remains difficult to distinguish acute myocarditis and myocardial infarction, particularly at an early stage. This patient was a rare case of *E coli*-induced myocarditis masquerading as acute STEMI and the first case with normal coronary arteries and infection-induced CAVB who became pacemaker dependent. The case highlights the importance of being aware of the etiologies of non-ischemia ST-segment elevation and the potential impact of *E coli* sepsis on the cardiac conduction system.

## Author contributions

**Conceptualization:** Po-Jen Hsiao, Ching-Han Liu, Yen-Lien Chou, Jun-Ting Liou.

**Data curation:** Ching-Han Liu, Yen-Lien Chou, Bo-Hau Chen.

**Formal analysis:** Po-Jen Hsiao.

**Funding acquisition:** Bo-Hau Chen.

**Investigation:** Po-Jen Hsiao, Bo-Hau Chen.

**Project administration:** Jun-Ting Liou.

**Supervision:** Jun-Ting Liou.

**Validation:** Po-Jen Hsiao, Ching-Han Liu, Jun-Ting Liou.

**Visualization:** Po-Jen Hsiao.

**Writing – original draft:** Ching-Tsai Hsu.

**Writing – review & editing:** Po-Jen Hsiao, Bo-Hau Chen.
